# Unilateral Proptosis as the Initial Manifestation of Acute Myeloid Leukemia in a Young Adult

**DOI:** 10.7759/cureus.72927

**Published:** 2024-11-03

**Authors:** Upamanyu Nath, Juhi Gupta, Chandana Chakraborti

**Affiliations:** 1 Ophthalmology, Nilratan Sircar Medical College and Hospital, Kolkata, IND; 2 Ophthalmology, Medical College and Hospital, Kolkata, Kolkata, IND; 3 Ophthalmology, Bankura Sammilani Medical College and Hospital, Bankura, IND

**Keywords:** acute myeloid leukaemia, chemotherapy, ocular chloroma, ocular manifestations, unilateral exophthalmos, unilateral proptosis

## Abstract

A 22-year-old male presented with progressive unilateral proptosis, conjunctival chemosis, and vision loss in the right eye over the course of one month, without associated systemic symptoms such as fever, fatigue, or infections. Initial imaging, including CT and MRI, suggested benign orbital conditions like cavernous hemangioma or orbital pseudotumor. Despite the absence of systemic signs, hematological investigations revealed anemia, thrombocytopenia, and leukocytosis. A peripheral blood smear raised suspicion of a hematologic malignancy, which was confirmed by bone marrow biopsy, diagnosing acute myeloid leukemia (AML) with the t(8;21) translocation, a favorable cytogenetic subtype associated with AML with maturation. The patient was promptly started on induction chemotherapy with cytarabine and daunorubicin, resulting in a significant reduction of proptosis, improved extraocular movement, and partial restoration of vision. This case illustrates the unusual presentation of AML with initial ocular involvement, a rare but critical manifestation, and emphasizes the importance of considering hematological malignancies in young patients presenting with unexplained proptosis. Multidisciplinary collaboration led to a timely diagnosis and effective treatment. Early recognition and prompt initiation of chemotherapy are essential for both systemic remission and ocular recovery in such atypical cases.

## Introduction

Proptosis, or exophthalmos, is defined as a 2 mm or greater asymmetry in the protrusion of a patient’s eyes [1]. It is a relatively uncommon clinical sign that can be attributed to a wide range of causes, both benign and malignant. Awareness of the causes of proptosis is essential, as many can lead to vision loss [2]. In cases of unilateral proptosis, common differential diagnoses include orbital cellulitis, developmental anomalies, inflammatory conditions, vascular anomalies, and neoplasms [3].

Proptosis, particularly in the absence of trauma or infection, is an uncommon presentation in systemic hematologic disorders such as acute myeloid leukemia (AML) [4]. AML is a rapidly progressing cancer of the blood and bone marrow, characterized by the proliferation of abnormal myeloid precursor cells [5]. It typically presents with systemic symptoms like fatigue, bleeding tendencies, or infections due to bone marrow failure. However, extramedullary manifestations, including ocular involvement, are less commonly seen and can complicate timely diagnosis. Ocular signs of AML, such as proptosis, occur when leukemic cells infiltrate the orbit, leading to a mass effect and displacement of the globe [4]. While leukemia-related proptosis is rare, it is crucial to consider this in the differential diagnosis, particularly when more common causes are absent.

Although this rare presentation has usually been reported in children [6-9], this case report describes a young adult who presented with unilateral proptosis, which was eventually diagnosed as an initial manifestation of acute myeloid leukemia. This case highlights the importance of considering malignancy in the differential diagnosis of proptosis, even in younger patients without systemic symptoms, and underscores the need for prompt evaluation and multidisciplinary management in such presentations.

## Case presentation

**Figure 1:**
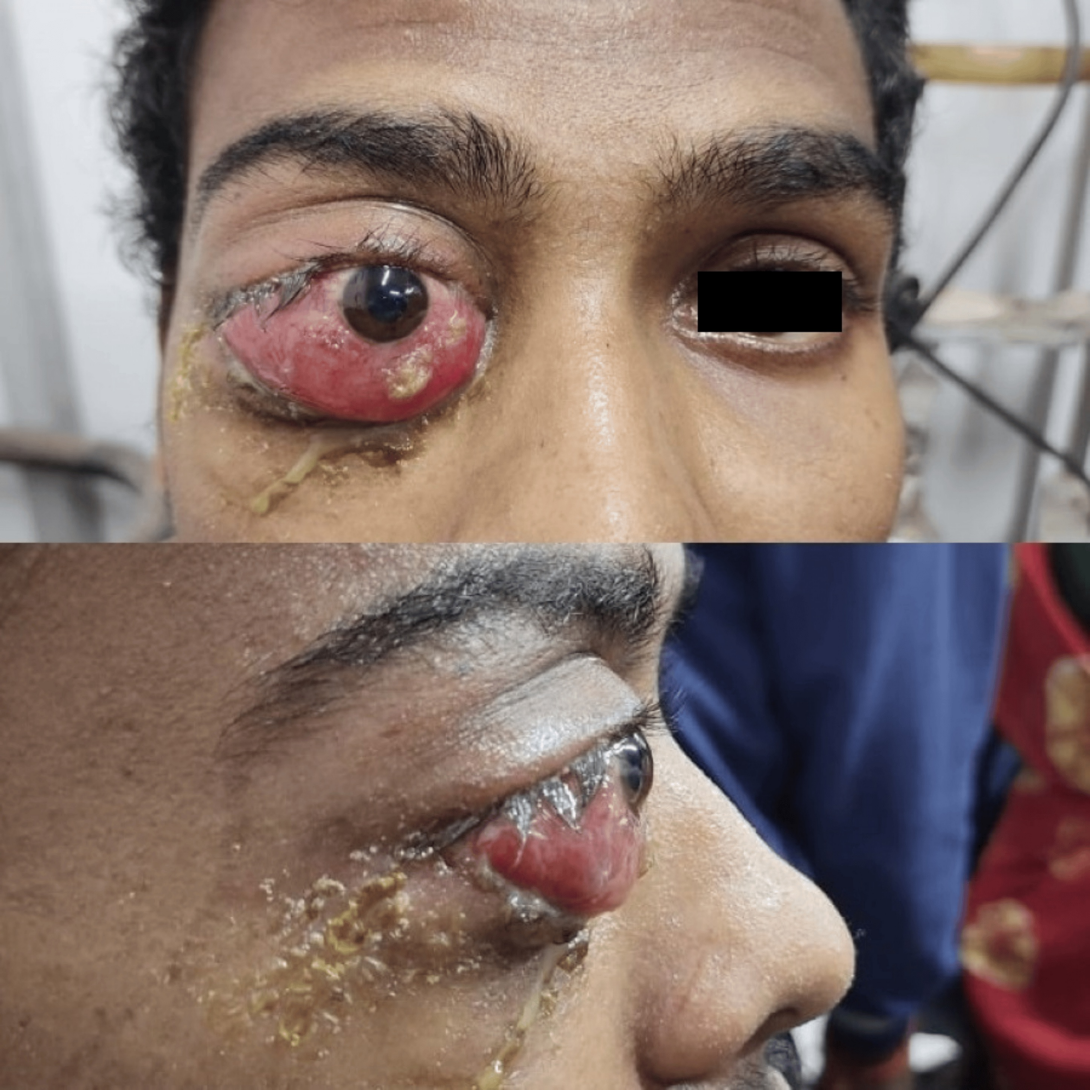
Initial presentation with proptosis

A 22-year-old male presented with progressive bulging and redness of the right eye (RE), accompanied by swelling of the upper and lower eyelids for the past month, as seen in Figure 1. He also reported a gradual decrease in vision in the right eye over the past 10 days. The patient did not experience any pain, diplopia, trauma, or systemic symptoms such as fever, headache, or neck swelling. He had no history of tremors, palpitations, or other systemic abnormalities, and there was no postural variation in the symptoms. The patient had no significant past medical history, including diabetes, hypertension, thyroid disease, tuberculosis, or malignancy. There was no history of previous ocular surgery or chronic medical conditions. His family history was unremarkable, with no known hereditary conditions or malignancies. 

The patient's general physical and systemic examination was within normal limits, with no evidence of lymphadenopathy or signs of systemic illness. On initial examination, visual acuity was 6/12 in the right eye (RE) and 6/9 in the left eye (LE). The external examination of the RE revealed marked conjunctival chemosis and congestion, along with mild corneal opacity secondary to exposure keratopathy attributed to lagophthalmos. Extraocular movements (EOM) in the RE showed total external ophthalmoplegia, while the LE exhibited full and unrestricted movement. There was significant axial proptosis in the RE, measuring 28 mm compared to 16 mm in the LE, determined using a ruler. The retropulsion test was positive for resistance, indicating a firm mass in the orbit. Further evaluation revealed a positive relative afferent pupillary defect (RAPD) in the RE, accompanied by disc edema on fundus examination. The patient also exhibited lagophthalmos in the RE, resulting in incomplete eyelid closure. On palpation, the orbital mass in the RE was firm but non-tender, with no detectable pulsation or increased temperature; the orbital rim was free from any palpable masses. Auscultation revealed no bruit over the orbital region, and regional lymph nodes were not palpable. The LE showed no abnormal findings on examination.

**Figure 2:**
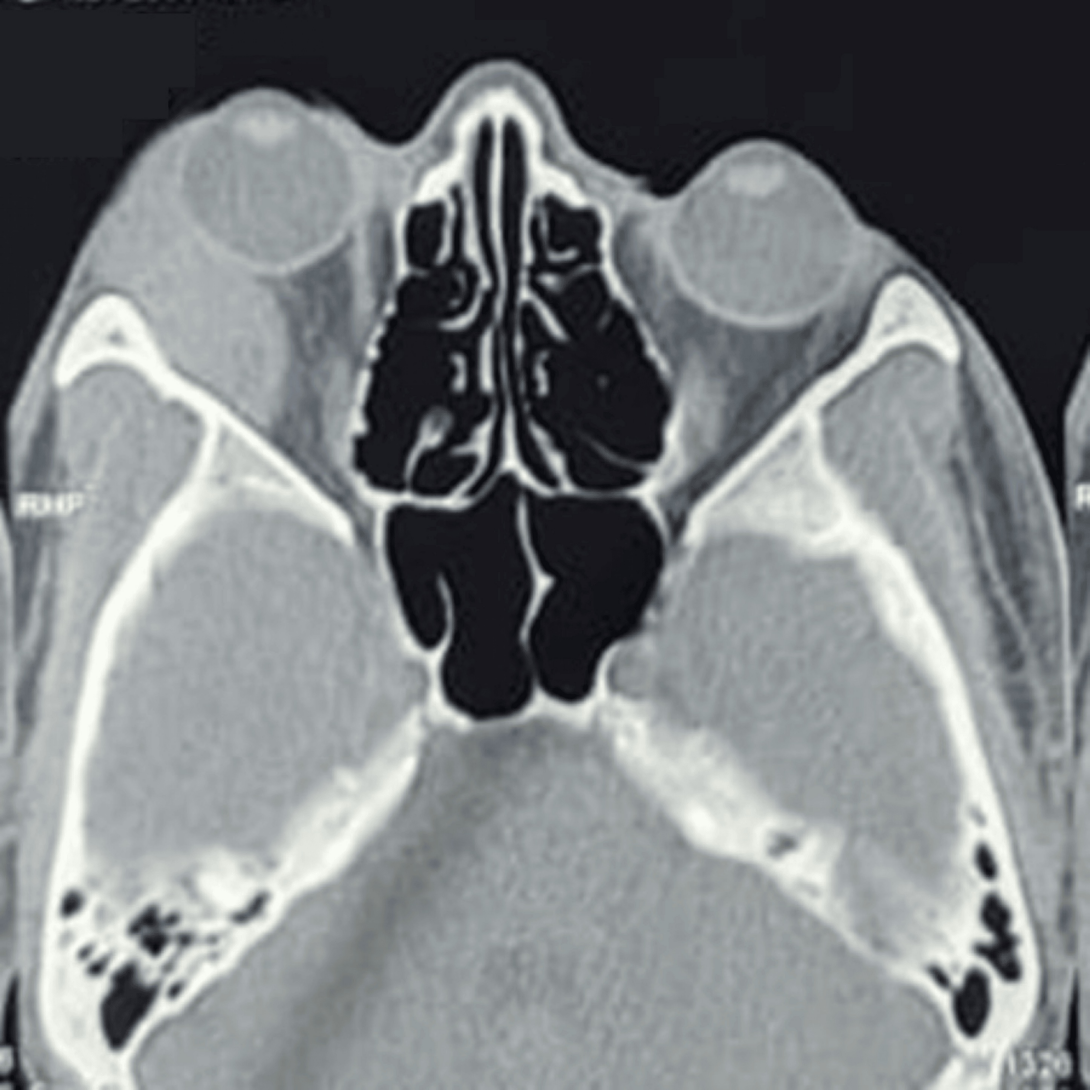
NCCT scan showing a soft tissue isodense lesion of size 33 x 12.2 x 33.5 mm3 in extra-conal space of right orbit on lateral aspect NCCT: Non-contrast computed tomography

**Figure 3:**
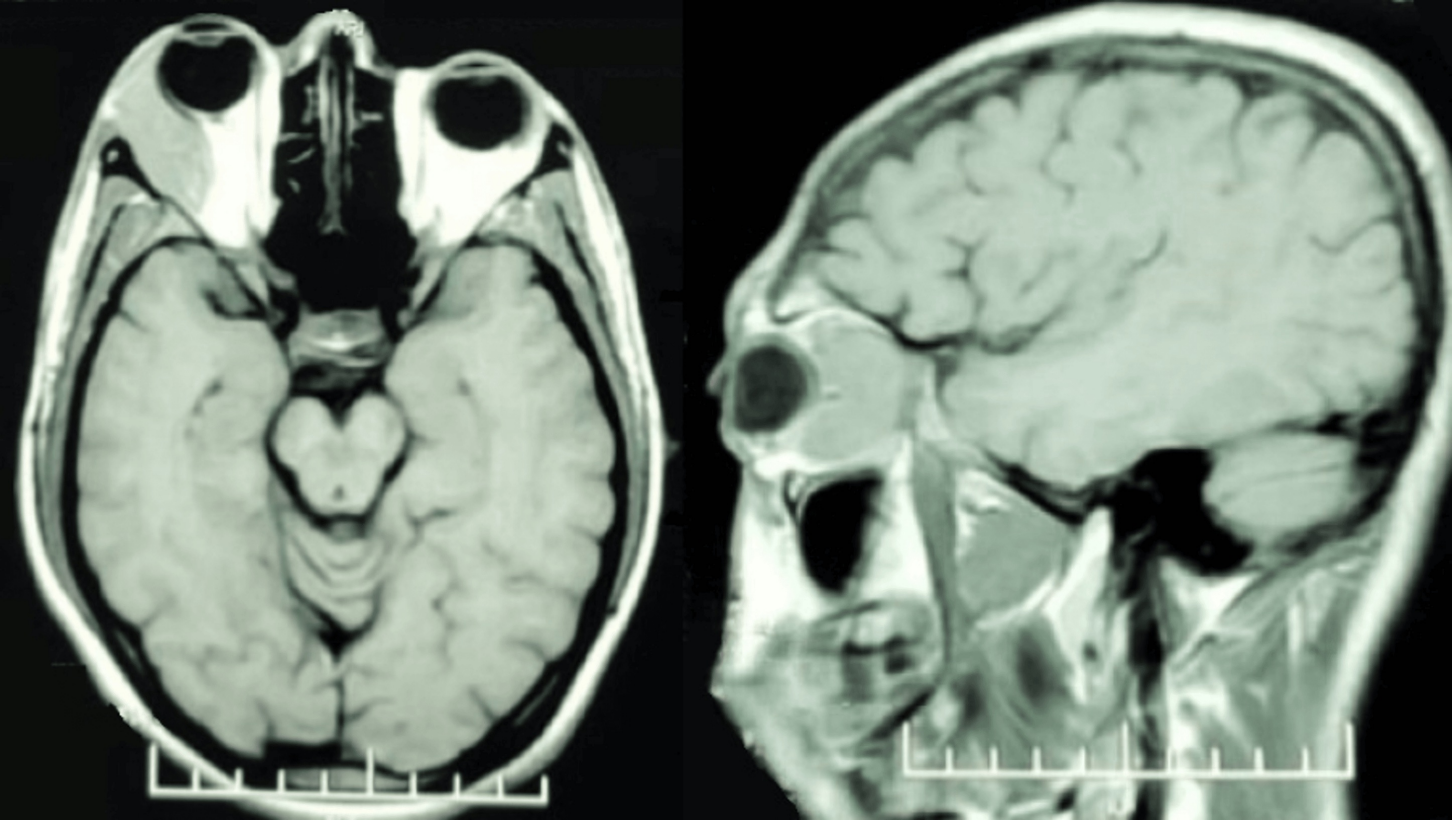
T1-weighted MRI showing the lesion behind the right eye

**Figure 4:**
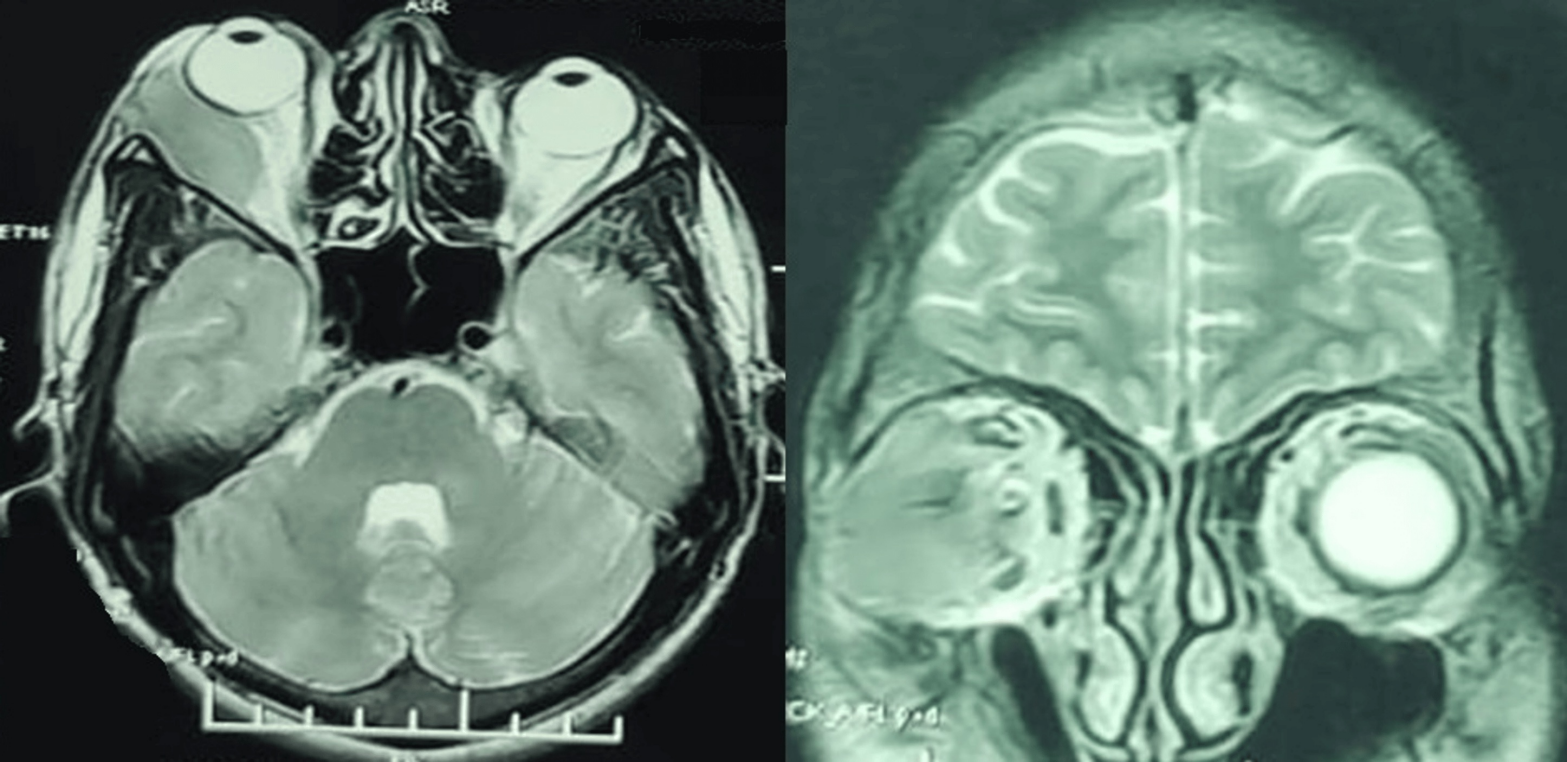
T2-weighted MRI showing isointense altered signal intensity lesion with diffusion restriction noted at lateral aspect of extraconal space of right orbit.

Initial neuroimaging with a non-contrast computed tomography (NCCT) scan of the orbit revealed a soft tissue isodense lesion measuring 33 x 12.2 x 33.5 mm in the extraconal space on the lateral aspect of the right orbit (Figure 2). This mass caused proptosis and medial displacement of the lateral rectus muscle. Differential diagnoses based on the NCCT findings included hemangioma, orbital pseudotumor, and dacryoadenitis, prompting a contrast study for further evaluation. Subsequent magnetic resonance imaging (MRI) of the orbit showed a hyperintense lesion on T2-weighted images with diffusion restriction, suggestive of a cavernous hemangioma in the lateral extraconal space (Figures 3, 4).

Hematological investigations revealed a hemoglobin level of 9.4 g/dL, a red blood cell count of 3.22 x 10⁶/μL, a white blood cell count of 19.5 x 10³/μL, and a platelet count of 55,000/μL. A peripheral blood smear showed normocytic, normochromic red blood cells with anisocytosis and the presence of atypical cells, raising suspicion for acute myeloid leukemia (AML). The differential count revealed 45% atypical cells, 36% neutrophils, 15% lymphocytes, 3% monocytes, and 1% eosinophils, with an elevated erythrocyte sedimentation rate (ESR) of 75 mm/hr. Biochemical analysis indicated an elevated serum lactate dehydrogenase (LDH) level of 1202 U/L and a fibrinogen level of 518 mg/dL, with normal kidney function and electrolytes. Coagulation studies, including prothrombin time (PT) and activated partial thromboplastin time (aPTT), were within normal limits.

Bone marrow examination confirmed the diagnosis of AML, showing the karyotype 46,XY,t(8;21)(q22;q22), a favorable karyotype associated with AML with maturation. Immunophenotyping demonstrated positivity for markers including MPO, CD13, CD33, CD117, and CD34, while markers such as CD11b, CD14, CD15, CD64, and lymphoid markers were negative. Molecular diagnostics revealed no mutations in the NPM1, FLT3, or CRBPA genes.

Based on clinical findings, imaging, and laboratory results, the patient was diagnosed with acute myeloid leukemia with maturation and a favorable karyotype (t(8;21)).

**Figure 5:**
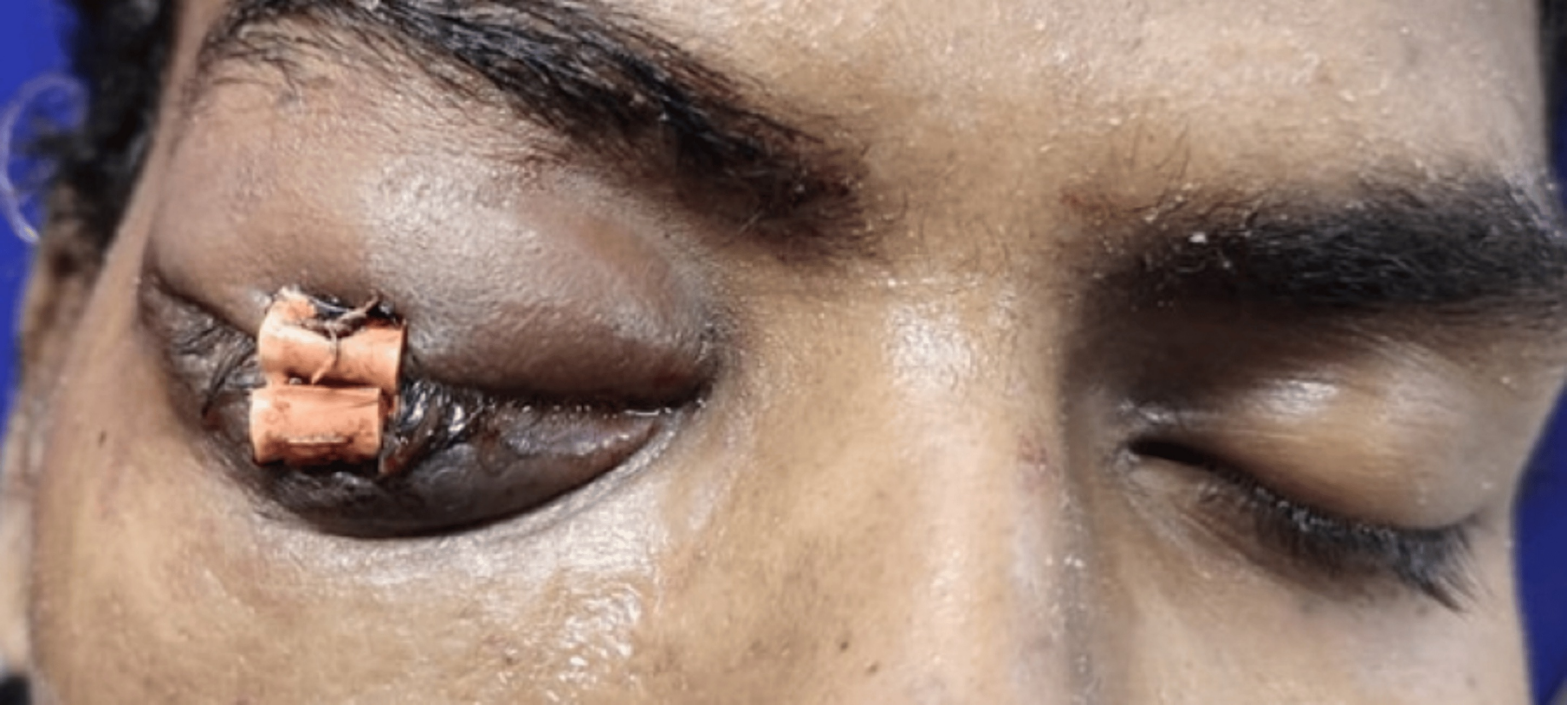
Tarsorrhaphy done on right eye for exposure keratopathy

**Figure 6:**
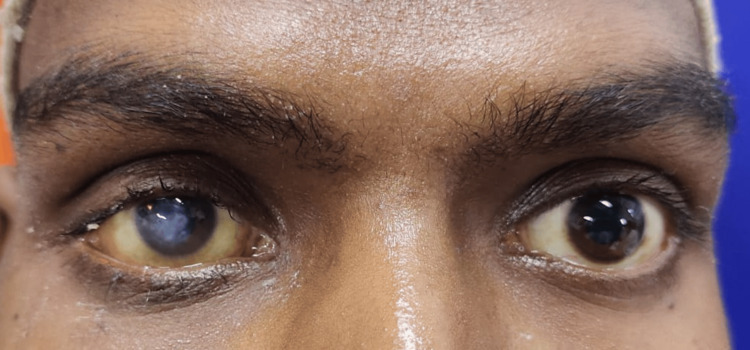
Exposure keratopathy in the right eye

The patient was promptly referred to the hematology department for further management. He was started on induction chemotherapy with a combination of cytosine arabinoside (100 mg/m²/day) IV (intravenous) for seven days and daunorubicin (60 mg/m²) IV for three days. This was followed by two cycles of intermediate-dose cytarabine as part of the consolidation therapy, with three such cycles administered at two-month intervals. Following the initiation of chemotherapy, there was a marked reduction in proptosis and chemosis. The patient's visual acuity improved to counting fingers at five feet in the RE and 6/6 in the LE. Although a tarsorrhaphy was performed (Figure 5), macular corneal opacity due to exposure keratopathy persisted (Figure 6). A penetrating keratoplasty (PKP) was planned, but the patient refused further surgery. Extraocular movements returned to full and unrestricted range in all directions, and fundus examination revealed a normal optic disc in both eyes.

## Discussion

Proptosis, especially in young adults, poses a unique diagnostic challenge due to its wide array of potential causes, ranging from benign orbital processes to life-threatening malignancies. The case presented illustrates the rare occurrence of acute myeloid leukemia (AML) manifesting initially as unilateral proptosis. This atypical presentation underscores the importance of a broad differential diagnosis and highlights the need for early recognition of hematological malignancies when common orbital disorders are excluded.

Orbital infiltration by leukemic cells, although rare - with most reports occurring in the pediatric population - is a recognized extramedullary manifestation of AML. Ocular involvement in leukemia is often secondary, occurring as part of a more widespread systemic disease [10]. However, in some cases, as in our patient, it can present as the initial clinical feature. The mechanisms underlying orbital involvement in AML include direct infiltration of leukemic cells into the orbit or, less commonly, compression due to localized chloromas, which are solid collections of myeloid blasts outside the bone marrow. Chloromas, also known as granulocytic sarcomas, can manifest in any part of the body but have a predilection for the orbit in rare cases, leading to proptosis, visual impairment, and restriction of extraocular movements [11].

In this case, the patient's presentation with proptosis, chemosis, and visual decline - without any systemic symptoms typically associated with leukemia, such as fatigue, pallor, or infections - delayed the suspicion of a hematological malignancy. The initial differential diagnoses of orbital pseudotumor, cavernous hemangioma, or dacryoadenitis were deemed more plausible based on clinical presentation and imaging findings. However, it was the peripheral blood smear and subsequent bone marrow biopsy that revealed the underlying hematological disorder. This emphasizes the critical role of laboratory investigations in cases of unexplained proptosis, especially when imaging alone does not lead to a definitive diagnosis.

AML is a heterogeneous disease with several cytogenetic subtypes, each carrying different prognostic implications. The patient in this case had the t(8;21) translocation, which is associated with AML with maturation, a subtype that typically responds well to chemotherapy and carries a relatively favorable prognosis. This genetic abnormality is commonly found in younger patients with AML and is linked to better overall survival rates compared to other AML subtypes [12]. The prompt initiation of induction chemotherapy in our patient led to a marked reduction in proptosis and improvement in visual acuity, aligning with the generally good response to treatment seen in AML with t(8;21).

The improvement in ocular symptoms following chemotherapy highlights the reversibility of leukemic infiltration with appropriate systemic treatment. While surgical intervention, such as decompression or biopsy, may sometimes be necessary to relieve orbital pressure or establish a diagnosis, our patient responded well to chemotherapy alone, suggesting that the primary treatment for ocular involvement in leukemia should be systemic. This case reinforces that ophthalmic manifestations of systemic diseases, including hematological malignancies, can improve significantly with appropriate medical therapy, potentially avoiding more invasive procedures.

## Conclusions

In summary, this case illustrates the rare but significant presentation of acute myeloid leukemia as proptosis in a young adult. It highlights the importance of maintaining a high index of suspicion for hematological malignancies in the differential diagnosis of orbital masses, particularly when more common etiologies are excluded by imaging and clinical evaluation. Prompt hematological investigation, leading to early diagnosis and chemotherapy, is crucial for achieving both systemic remission and ocular recovery. This case emphasizes the need for comprehensive systemic workup in patients with unexplained proptosis and reminds clinicians of the broad differential diagnosis that includes life-threatening conditions such as AML.
